# The Gift of Expression

**Published:** 1920-10-02

**Authors:** 


### THE EDITOR'S LETTER-BOX.

**Correspondence on all subjects is invited, but we cannot in any way be responsible for the opinions expressed by our correspondents, who must give their name and address as a guarantee of good faith, but not necessarily for publication. Correspondents are reminded that brevity of style and conciseness of statement greatly facilitate early insertion.**

### The Gift of Expression.

*To the Editor of* THE HOSPITAL.

SIR,—If the effect of the curricula of the nurses' training-schools, in the characters of the women they turn out, is to render them plastic and easily moulded, then indeed those in authority have failed, and failed miserably, in their standard of ethics. In the nursing services to-day we need women of sound judgment and clarity of thought.

It is our English pride that we govern by the will of the people, a principle we apply to our professional life. It is the will of the rank and file that elects the leaders; it is the interests of the rank and file those leaders are pledged to protect; it is the subscriptions of the rank and file that defray their expenses; and it is the existence of the rank and file that gives them place and power.

No leader can work for professional welfare unless she knows the temper of the rank and file, and only by ample discussion can she gauge the depth and the breadth of the professional mind.

The nurses of to-day must accept their responsibilities, they must shake off their apathy, their indifference, they must learn to know themselves or they must cease to exist, and if their condition is already so enfeebled that it will not bear comment in the columns of the nursing Press, then their state is parlous indeed, and no "shouting from house-tops" can save them.—Yours faithfully,

September 27. THE WRITER OF THE ARTICLE.

